# Metaplastic papillary tumour of the fallopian tube, a rare entity, analysed by next‐generation sequencing

**DOI:** 10.1111/his.14043

**Published:** 2020-03-24

**Authors:** Sandra Sunitsch, Julia Reisinger, Luca Abete, Karl Kashofer, Peter Regitnig

**Affiliations:** ^1^ Diagnostic and Research Institute of Pathology Medical University of Graz Graz Austria; ^2^ Department of Obstetrics and Gynecology Medical University of Graz Graz Austria

**Keywords:** metaplastic papillary tumour, fallopian tube, next‐generation sequencing

*Sir*: Metaplastic papillary tumour of the fallopian tube (MPT) is an extremely rare lesion. To the best of our knowledge, only 12 cases of MPT have been documented in the literature to date, usually detected incidentally upon examination of fallopian tube segments removed for sterilisation postpartum. Only one case has been reported to be unrelated to pregnancy.^1^

Debate exists as to whether this lesion represents a true neoplasm or a metaplastic proliferative lesion.^2^, ^3^ So far, molecular studies have only referred to *KRAS* and *BRAF*,^1^ as well as to microsatellite analysis.^3^

Herein, we report the first case of a 35‐year‐old woman with an MPT, which has been analysed by next‐generation sequencing (NGS). Ethical approval for this study was obtained from the Ethikkommission Medizinische Universität Graz (Votum32‐045 ex 19/20) on 31 October 2019.

The fallopian tubes of our reported patient were removed postpartum for sterilisation. Macroscopic examination did not detect any abnormalities. The tissue samples were routinely processed and stained by haematoxylin and eosin staining (H&E), according to our standard protocol.

Histological examination revealed an exophytic lesion within the tubal lumen of the right salpinx measuring 1 mm in its greatest dimension, involving only part of the mucosa (Figure 1A). The proliferation showed a papillary configuration with loose fibrovascular connective tissue (Figure 1B). The epithelial lining consisted of one or two layers of plump, non‐ciliated cuboidal cells with eosinophilic cytoplasm mainly in a pseudopapillary or papillary architecture (Figure 1C). The nuclei of these cells were centrally located, round or oval. In a few areas, however, the nuclei displayed a variable appearance, with either dense or vesicular chromatin and small nucleoli. Mitoses or apoptoses were absent. Additionally, scant extracellular mucin was observed. Some intraluminal tufts included fibrovascular cores containing small blood vessels. The lesion showed a sparse lymphocytic infiltration (Figure 1B,C).

**Figure 1:**
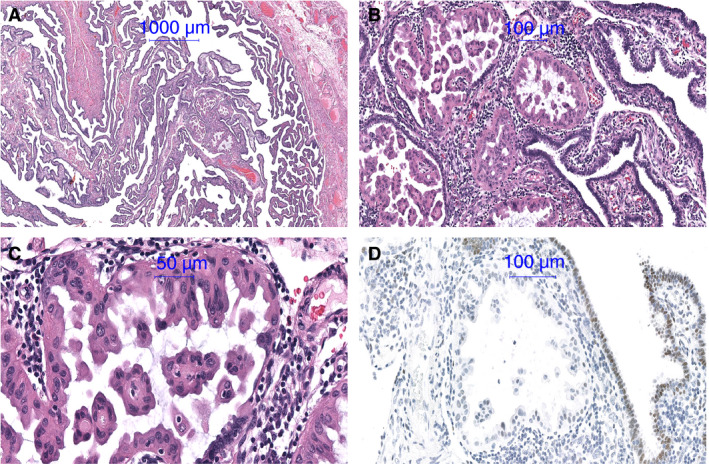
**A**, Metaplastic papillary tumour (MPT) involving a small part of the mucosa. **B**, Papillary configuration with loose fibrovascular connective tissue and sparse lymphocytic infiltrate. **C**, Epithelial lining consists of one or two layers of plump, non‐ciliated columnar cells with eosinophilic cytoplasm and stroma with sparse lymphocytic infiltrate. **D**, Androgen immunoreactivity of MPT cells was negative compared to that of normal tubal epithelium.

Due to the eosinophilic or apocrine‐like morphology, immunohistochemistry (IHC) with an antibody against androgen receptor was performed on a Dako Autostainer, but otherwise, due to limited material, molecular diagnostics were conducted instead of immunohistochemistry. After manual microdissection, mutational analysis was performed using the Ion Torrent Comprehensive Cancer Panel (cat. no.:4477685; Life Tech Austria, Vienna, Austria), covering 409 genes frequently implicated in cancer, accompanied by low‐density whole‐genome sequencing to detect copy number variations.

Androgen receptor immunoreactivity of the eosinophilic cells was reduced compared to the slightly positive normal tubal epithelium (Figure 1D). No somatic mutations were found in the coding sequence of the 409 genes analysed, and no copy number alterations could be detected in the genome of this lesion.

The differential diagnoses considered included serous borderline tumour (SBT), serous tubal intra‐epithelial carcinoma (STIC) or low‐grade serous carcinoma, as follows.

- Serous borderline tumour of the fallopian tube is an extremely rare lesion, which resembles the ovarian counterpart, showing a hierarchical branching pattern with irregular papillae, branching from large to smaller papillae. The lining epithelium consists of non‐stratified and stratified cuboidal to columnar cells. The tumour cells are polygonal or hobnail‐like with eosinophilic cytoplasm and moderately enlarged nuclei. Borderline tumours of the ovary, spreading to the tube are more likely than borderline tumours of the tube. These lesions harbour *KRAS* and *BRAF* mutations.^4^, ^5^
- Most STICs are found in the distal tube, particularly in women with *BRCA1* or *BRCA2* mutation which confers a high risk of developing this neoplasm. The neoplastic epithelium shows pleomorphic stratified and non‐ciliated cells with an increased nuclear‐cytoplasmic ratio and loss of polarity; 92% of STIC show *TP53* mutations.^5^
- Low‐grade serous carcinoma of the fallopian tube is morphologically identical to its ovarian counterparts and is characterised by glands haphazardly infiltrating the stroma. It is usually associated with serous borderline tumour and harbours *KRAS* and *BRAF* mutations in 50–60% of cases.^5^

The fact that the reported cases of MPT in the literature with available follow‐up information showed no recurrence of or death due to tumour,^1^ and that in addition we could not demonstrate any copy number variation or any mutation in a 409 gene panel of cancer‐related genes, suggests the metaplastic nature of MPT.

Hence, we emphasise the importance of not misdiagnosing this entity as serous borderline tumour, serous tubal intraepithelial carcinoma or low‐grade serous carcinoma to avoid overtreatment.

## Conflicts of interest

The authors state that they have no conflicts of interest.

## References

[1] Jang MI , Sung JY , Kim JY , Kim HS . Clinicopathological characteristics of metaplastic papillary tumor of the fallopian tube. Anticancer Res. 2017; 37; 3693–3701. 28668862 10.21873/anticanres.11741

[2] Saffos RO , Rhatigan RM , Scully RE . Metaplastic papillary tumor of the fallopian tube – a distinctive lesion of pregnancy. Am. J. Clin. Pathol. 1980; 74; 232–236. 7405904 10.1093/ajcp/74.2.232

[3] D'Adda T , Pizzi S , Bottarelli L , Azzoni C , Manni S , Giordano G . Metaplastic papillary tumor of the salpinx: report of a case using microsatellite analysis. Int. J. Gynecol. Pathol. 2011; 30; 532–535. 21979587 10.1097/PGP.0b013e31821713d2

[4] Salazar MF , Moscoso IE , Vazquez LT , Lopez Garcia NL , Escalante Abril PA . Fallopian metaplastic papillary tumour: an atypical transdifferentiation of the tubal epithelium? J. Pathol. Trans. Med. 2015; 49; 148–155. 10.4132/jptm.2014.10.15 PMC4367111 25812736

[5] Kurman RJ , Carangiu ML , Herrington CS , Young RH . WHO classification of tumours of female reproductive organs. 4th ed Lyon: IARC, 2014.

